# Biosocial Strategies to Address the Socioeconomic Determinants and Consequences of the TB and COVID-19 Pandemics

**DOI:** 10.4269/ajtmh.20-1641

**Published:** 2021-01-06

**Authors:** Debora Pedrazzoli, Tom Wingfield

**Affiliations:** 1 Department of Infectious Disease Epidemiology, London School of Hygiene and Tropical Medicine, London, United Kingdom;; 2 LIV-TB Lead, Departments of Clinical Sciences and International Public Health, Liverpool School of Tropical Medicine, Liverpool, United Kingdom;; 3 Tropical and Infectious Disease Unit, Liverpool University Hospitals NHS Foundation Trust, Liverpool, United Kingdom;; 4 Department of Global Public Health, Karolinska Institutet, Stockholm, Sweden

COVID-19 and tuberculosis (TB) are damaging, dual pandemics, which are more than mere health crises; they are socioeconomic and humanitarian crises that require a biosocial response.^1,2^

The socioeconomic determinants of TB and COVID-19 are pernicious and overlapping. Poverty, overcrowded housing conditions, under- or malnutrition, chronic comorbidities such as lung disease and diabetes, and belonging to marginalized, underserved communities and minority ethnic groups are all key determinants.^3^ COVID-19 has distorted health systems at all levels. It has contracted clinical services, decimated staffing levels, reconfigured laboratories including the repurposing of GeneXpert modules for TB diagnosis, rolled back hard won progress toward Universal Health Coverage (UHC) and global health security,^4^ and diverted much-needed resources away from other diseases, including TB.^5,6^ As a result, the COVID-19 pandemic is predicted to lead to a 20% increase in TB deaths in high-burden countries over the next 5 years.^7^

The health and socioeconomic consequences of TB and COVID-19 are highly harmful and inequitably distributed.^8^ Impoverished individuals, households, and communities continue to be disproportionately affected by both TB and COVID-19. Social distancing and isolation measures, restricted movement and quarantine, illness, and care-seeking impose a severe socioeconomic burden, especially on those who are unemployed, in the informal job sector, or lack adequate social protection.^1^ These factors are not only associated with impaired healthcare access and worse health outcomes^9,10^; they can push those affected into further impoverishment, typifying the “medical poverty trap.”^11^ The corrosive influence of COVID-19 and its related mitigation strategies on mental health and domestic violence is a parallel, concomitant emergency.^12^

Far from being great “levellers,” these intersecting pandemics have reemphasized intolerable and persistent global inequalities in health, wealth, and well-being—inequalities that are aggravated by poverty of voice, agency, and opportunity.^13^ However, the convergent challenges brought about by COVID-19 and TB enable us to identify potential opportunities to mitigate their impact.

Key to this is collaboration toward achieving the interlaced WHO Sustainable Development Goals (SDGs)—specifically SDG1 (No poverty), SDG2 (Zero hunger), SDG3 (Good health and well-being), SDG8 (Decent work and economic growth), and SDG10 (Reducing inequalities).^14^ Modeling studies have suggested that eradicating poverty and providing social protection would reduce TB incidence by more than 84% by 2035^15^ – similar modeling studies would be of benefit when applied to COVID-19. Moreover, in the WHO End TB Strategy—for the first time in the modern era of TB control—we were provided with an ambitious socioeconomic goal that “zero TB-affected families face catastrophic costs” by 2025.^16^ In reality, catastrophic costs mitigation should be a global goal regardless for all states of ill health, whether mental or physical, noncommunicable or communicable, occupational or accidental, COVID-19 or TB.

The article by Fuady et al.^17^ in this issue of the *American Journal of Tropical Medicine and Hygiene* examining the impact of COVID-19 on costs incurred by people with TB and their households is topical, timely, and moves this field forward. The article draws attention to this overlooked area of research, policy, and practice, and provides an informative conceptual framework that will help us to develop better biosocial responses to combat both TB and COVID-19.

So, how can we address the socioeconomic determinants and consequences of TB and COVID-19, and prevent what our colleagues have rightly called the “perfect storm?”^18^

First, there is an urgent need to better understand the socioeconomic impacts of COVID-19, especially in the most vulnerable groups. Evidence from nationally representative surveys of costs incurred by people with TB and their households (known as “TB Patient Cost Surveys”) shows that more than one in two TB-affected households worldwide incur catastrophic costs.^19^ These surveys and related mixed-methods research also show that significant drivers of catastrophic costs are: lost income and time, reduced productivity, and significant non-medical expenses such as travel to access healthcare services and nutritional expenditure to meet even basic food requirements.^20–22^ In certain regions, these costs may be exacerbated by an unregulated and convoluted public–private sector mix^23^ and avoidable expenditure on unproven, costly “therapies” including nutritional supplements. Furthermore, mask-wearing and respiratory symptoms of COVID-19 and TB may be associated with diagnostic uncertainty, stigma, discrimination, and isolation, which further compound challenges to accessing care and appropriate treatment.^18,24^ We have the perfect opportunity now to measure and evaluate the socioeconomic impacts along the care-seeking pathway for people with acute, chronic, or acute-on-chronic respiratory symptoms.

Second, understanding the patient pathway will provide valuable information to inform assessment of the progress made toward the SDGs. This is particularly pertinent with relation to achieving SDG3 and UHC but also to the under-acknowledged yet acute need to expand social protection coverage and establish basic universal social protection floors.^25,26^ National lockdown, “stay at home,” and “shelter in place” policies have already had a detrimental effect on individual and household incomes and been associated with contractions in national gross domestic product indices.^14^ There is a clear negative association between incidence and mortality of TB and proportional governmental expenditure on social protection.^27,28^ It is as yet unclear whether the same association exists with relation to COVID-19, but that evidence will be vital to shape our response.

Third, despite their detrimental effects, large-scale non-pharmacological interventions may also provide a useful opportunity to explore ways in which we can mitigate care-seeking costs of TB, COVID-19, and other poverty-related diseases. For example, measures that reduce the need for daily encounters between TB patients and healthcare staff to reduce COVID-19 transmission have been rapidly rolled out and could be further strengthened.^29^ One such measure is the WHO recommendation of all-oral TB treatment regimens for multidrug- and rifampicin-resistant TB and extensively drug-resistant TB.^30^ This strategy can lower costs for people with TB and their households through reduction of direct costs associated with traveling to health facilities for daily injections, and/or opportunity costs associated with lost productivity and income due to hospitalization during the initial “intensive” phase of treatment.^31^ Similarly, intensified use of digital health technologies, such as video observed therapy and electronic medication monitors to support programs and affected people could pave the way to models of care that may lower medical, non-medical, and opportunity costs even further.^32^

Fourth, the COVID-19 pandemic has brought into sharp relief the weaknesses of health systems that are imbalanced—in terms of both funding and focus—toward secondary care. We could use this opportunity to garner feedback from users of the health system on how best to improve the care we provide not only in secondary care but at all levels.^33^ This is also an opportune moment to reconsider how best to engage communities and community health workers to harness their commitment, knowledge, trust, and understanding of their local areas.^34^ Outreach involving peer support and advocacy is a much needed vehicle of change to not only reduce care-seeking costs by bringing care to people’s door in the community but also to use peer-led education and information as a tool to promote agency, to empower, and to combat stigma.^35^ In this way, we could ensure no one is left behind and eradicate the misplaced blame attributed to the so-called “hard-to-reach” groups who, in reality, are underserved by static and fragmented local health and social care systems.^36^

Finally, building on the aforementioned, this is the time to move away from vertical public health programs. We need health systems that are responsive to the needs of vulnerable communities and resilient to the threat of infections, especially those which are airborne. A major step toward reaching this goal would be inclusive horizontal approaches that integrate biomedical strategies to prevent and tackle the health impacts of syndemics with biosocial strategies to address their determinants and consequences.^37^ People affected by poverty-related illnesses, including respiratory illnesses, would benefit vastly from this joined-up approach.

The COVID-19 pandemic is threatening decades of progress made in TB control and, undoubtedly, continues to have the biggest impact on people and communities affected by TB and poverty. Predicted increases in levels of food insecurity, rising impoverishment, and contraction of gross domestic product have led to cross-sectoral bodies, including the WHO, academia, and civil society, to ring the alarm bell.^38^ Findings from ongoing and future TB Patient Cost Surveys will provide an accurate estimate of the socioeconomic impact of COVID-19 on people with TB and their households. The surveys will simultaneously provide critical data to inform broader mapping of social determinants and consequences of ill health, uptake and coverage of social protection strategies and UHC, and therefore progress toward the SDGs.

As we begin what we hope will be a brighter 2021, we must turn the challenges of the COVID-19 and TB pandemics into an opportunity to refine biosocial strategies that not only improve health and well-being but also address poverty and inequality.
